# Effect of Suberoylanilide Hydroxamic Acid and Phytosulfokine-Alpha on Successful Plant Regeneration from Embryogenic Callus-Derived Protoplasts of Garlic (*Allium sativum* L.)

**DOI:** 10.3390/ijms27010254

**Published:** 2025-12-25

**Authors:** Katarzyna Stelmach-Wityk, Kamil Szymonik, Dariusz Kadluczka, Iwona Jedrzejczyk, Ewa Grzebelus

**Affiliations:** 1 Department of Plant Biology and Biotechnology, Faculty of Biotechnology and Horticulture, University of Agriculture in Krakow, Mickiewicza 21, 31-120 Krakow, Poland; k.stelmach@urk.edu.pl (K.S.-W.); k.szymonik@urk.edu.pl (K.S.); darek.kadluczka@gmail.com (D.K.); 2 Department of Biotechnology, Bydgoszcz University of Science and Technology, Kaliskiego Ave. 7, 85-796 Bydgoszcz, Poland; jedrzej@pbs.edu.pl

**Keywords:** bulb crops, crop improvement, deacetylation inhibition, suberoylanilide hydroxamic acid, phytosulfokine, epigenetic modifications, protoplast-to-plant regeneration, somatic embryogenesis, tissue cultures, vorinostat

## Abstract

Garlic’s vegetative reproduction limits genetic improvement, necessitating advanced biotechnological tools like protoplast culture. However, efficient protoplast regeneration in monocots such as garlic remains a significant challenge. This study establishes an optimized protocol for embryogenic callus induction and subsequent protoplast-to-plant regeneration in garlic (*Allium sativum* L.), aiming to overcome current limitations using suberoylanilide hydroxamic acid (SAHA), a histone deacetylase inhibitor, and phytosulfokine-alpha (PSK). We successfully induced embryogenic callus from four garlic accessions and refined protoplast isolation and culture conditions. Key optimizations included using a specific enzyme mixture (2% cellulase R-10 and 0.2% pectolyase Y23) for high yields (from 0.8 to 2.1 × 10^6^ protoplasts per g FM) of viable (approx. 90%) protoplasts and employing the enriched K8M culture medium. Short exposure of protoplasts to SAHA (0.05 or 0.1 µM) significantly improved microcallus formation and plant regeneration. Notably, only callus derived from SAHA-treated cultures displayed regeneration potential, highlighting its pivotal role in embryo differentiation and development. This optimized protocol achieved a 70% success rate for plant acclimatization to ex vitro conditions, with 97% of regenerated plants retaining the ploidy of the donor accession. We demonstrate that SAHA and PSK application enhances garlic protoplast regeneration efficiency. This reliable system provides the groundwork for advanced biotechnological applications, including gene editing technologies in garlic.

## 1. Introduction

Garlic (*Allium sativum* L.) is a widely cultivated bulb crop and holds significant importance in global agriculture and culinary traditions [1]. Originating in Central Asia, this species has become extensively cultivated and globally utilized for both its culinary and traditional medicinal properties [2]. Garlic is reproduced vegetatively, which can limit genetic improvement and disease resistance in this crop. The inability of garlic to reproduce sexually presents considerable challenges, limiting not only genetic improvement but also directly affecting the cost of production. This is primarily due to reliance on costly vegetative propagules for its cultivation, a practice that simultaneously facilitates disease transmission [3]. Consequently, the establishment of an efficient method for the large-scale propagation of garlic is needed. Tissue culture protocols are efficient tools for complementing traditional garlic breeding programs and producing new cultivars [3].

To date, multiple micropropagation approaches have been successfully implemented in garlic breeding. These include meristem cultures (e.g., stem discs [4,5], shoot and root tip cultures [6,7]), somatic embryogenesis [8,9,10], and protoplast cultures [11], each offering distinct advantages for clonal propagation [12]. Protoplasts offer significant advantages for genetic engineering and subsequent regeneration into complete plants [13]. Their ability to develop into whole plants from single, isolated cells makes them an ideal system for genomic modifications, such as targeted gene editing using CRISPR-Cas or the creation of somatic hybrids [14,15].

To date, the use of protoplast technology in garlic has been scarce and limited mostly to the establishment of initial isolation and culture protocols [11,16], and the potential use of isolated protoplasts for somatic hybridization of two garlic cultivars [17] and of onion and garlic [18]. Yet, achieving high isolation efficiency and robust plant regeneration from protoplasts remains a considerable challenge, consistent with the general view that monocots are particularly recalcitrant to protoplast technology and protoplast-to-plant regeneration [19,20]. Despite the promise, persistent challenges in garlic protoplast cultures, particularly regarding sustained divisions of protoplast-derived cells, continue to obstruct the routine application of this technology across diverse garlic cultivars and landraces. Ayabe and Sumi [11] reported a callus formation frequency of only 0.04% to 5%, whereas the study of Hasegawa et al. [16] showed a frequency of 2.2%. The source tissue used for protoplast isolation can play a crucial role in obtaining high yields of good-quality protoplasts. The employment of callus, especially of an embryogenic nature, can improve regeneration success when the regenerative capacity of protoplasts derived from somatic cells is low [21,22]. In many species, the development of protoplast culture can be ensured by additional supplements, such as polyamines [23,24] or inhibitors of phenolic compounds [24,25,26]. An example of peptide growth factor application is phytosulfokine-alpha (PSK), a sulfated pentapeptide promoting cell growth and proliferation [27]. PSK has been reported to enhance protoplast cultures of many industrial and crop species [24,28,29,30,31,32,33,34,35].

One key factor often limiting the successful plant regeneration from protoplasts is the presence of endogenous cellular mechanisms that regulate cell cycle progression and cellular differentiation, often leading to cellular senescence or aberrant development [36]. DNA methylation and histone deacetylation are critical epigenetic mechanisms influencing gene expression, and their modulation has been shown to significantly impact plant regeneration efficiency in various species [37,38,39].

Research on somatic embryos and microspores indicates that induced epigenetic modifications can influence the expression of genes crucial for developmental processes [40,41,42]. While studies on protoplasts in this area are limited, existing findings imply that cellular competence might be regulated through alterations in DNA methylation and/or histone deacetylation [43,44]. In many species, it has been found that initial stages of cell reprogramming and potential embryogenesis initiation usually involve DNA hypomethylation [45,46], acetylation of histones H3 and H4 [47], and demethylation of histone H3K9 [47,48]. Inhibitors of histone deacetylases, such as trichostatin A (TSA) or suberoylanilide hydroxamic acid (SAHA; vorinostat), have been shown to promote totipotency of male gametophytes of *Brassica napus* L., *B. rapa* ssp. *chinensis* L. and *B. oleracea* var. *acephala* L. [41,49,50], and to trigger somatic embryogenesis transition in *Arabidopsis thaliana* [51].

Here, we present the protocol for embryogenic callus induction and somatic embryogenesis-mediated plant regeneration in garlic. Additionally, we optimize the protocol for callus-derived protoplast isolation, culture, and plant regeneration. We explore the effect of culture media composition and the supplementation of PSK and SAHA on overcoming division latency and promoting cell divisions in protoplast cultures. These factors also facilitate proembryogenic callus formation, somatic embryogenesis, and subsequently plant regeneration from protoplast-derived cells.

## 2. Results

### 2.1. Induction and Culture of Clove-Derived Callus

**Figure 1:**
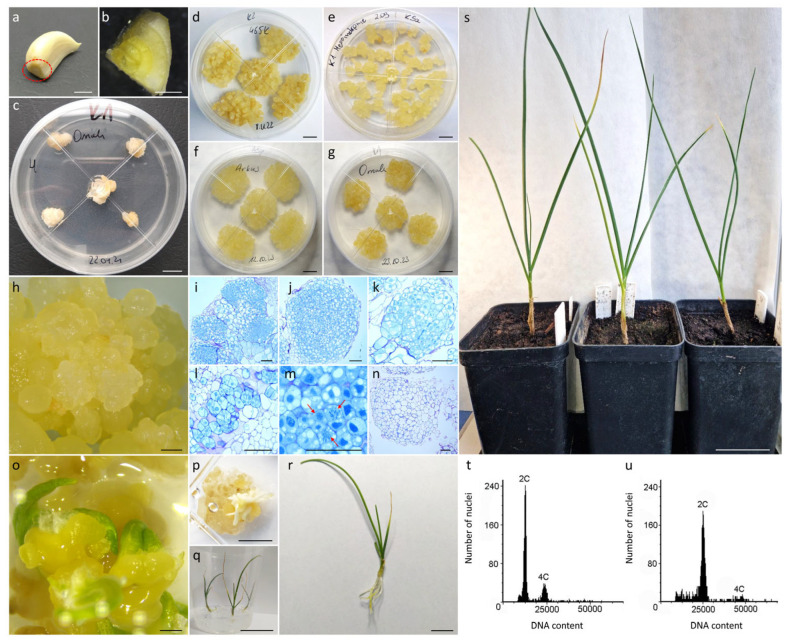
Callus induction from the base of garlic cloves and plant regeneration. (**a**) Garlic clove base indicated by a dotted line; (**b**) piece of clove base with true stem; (**c**) one-month-old callus culture; (**d**–**g**) stable eight-month-old callus cultures maintained on K1 medium; (**h**) friable callus with globular structures of various sizes; histological sections of friable callus with distinguishable embryogenic zones composed of small-sized, compact cells (**i**–**l**) with dense cytoplasm and large, dark blue-stained nuclei or (**m**) undergoing mitosis (pointed with red arrows) and (**n**) nonembryogenic zones; somatic embryos development (**p**) one and (**o**) three months after transferring friable, proembryogenic callus to hormone-free ½ BDS medium and light; (**q**) completely regenerated callus-derived plantlets; (**r**) in vitro regenerated plantlet ready for acclimatization to ex vitro conditions; (**s**) eight-month-old acclimatized garlic plants; exemplary histograms of relative nuclear DNA content for (**t**) diploid and (**u**) tetraploid in vitro regenerated garlic plant. Scale bars: 5 cm (**q**,**s**); 1 cm (**a**–**g**,**p**,**r**); 1 mm (**h**,**o**); 100 µm (**i**–**n**).

All four of the studied garlic accessions formed callus (Figure 1a–c) on the base of the clove within eight weeks of culture on both induction media (Table 1, Figure 1d–g). The frequency of callus formation varied significantly (*p* ≤ 0.05; Table 2; Supplementary File S1: Table S1) between accessions and ranged from 15.3% (‘Messidrome’) to 85.4% (465K). The formed callus was of either dry (friable; Figure 1d–h) or watery structure. In most accessions, the formed callus was predominantly friable, with the exception of 465K, where the composition of callus induction medium substantially impacted the structure of the formed callus (Table 1). All 465K explants cultured on K1 medium formed watery callus, whereas explants cultured on K2 medium exclusively formed friable callus (Figure 1d). The callus induction medium used did not impact the intensity of callus formation (*p* > 0.05) but affected the formation of friable callus (Table 2; Supplementary file S1: Table S2), generally showing higher levels of friable callus formation on K2 medium. No significant differences in the levels of embryogenic callus formation (*p* > 0.05) were observed with respect to either the analyzed accessions, or the callus induction media (Table 2). After 12 weeks of culture, stable lines of pale yellow embryogenic callus were obtained for all four accessions. To ensure continued growth and maintenance of stable callus lines, K1 medium was selected. This medium enables the rapid propagation of material after each passage (within 12–16 days), thereby ensuring sufficient material of suitable age for protoplast isolation. Only the friable embryogenic callus was transferred onto fresh K1 medium every 3–4 weeks (Figure 1d–g).

### 2.2. Histological Analysis of Clove-Derived Callus and Plant Regeneration

Based on macroscopic observations, friable callus, regardless of the callus induction medium, was composed of globular structures of various sizes (Figure 1d,h). Microscopic observations of histological sections from both callus induction media revealed that these globular structures consisted mostly of distinguishable embryogenic zones (Figure 1i–m), but nonembryogenic zones were also present (Figure 1n). In contrast to the large, vacuolated cells of the nonembryogenic mass, the embryogenic clusters were composed of small-sized, compact cells of isodiametric shape, with dense cytoplasm, numerous small vacuoles and large, round-shaped, dark blue-stained nuclei, indicating their meristematic nature (Figure 1l). Additionally, cells undergoing mitosis were often observed in these zones (Figure 1m). Such multicellular meristematic masses gave rise to proembryogenic masses containing pre- or globular-like somatic embryos (Figure 1h). After transferring proembryogenic mass onto ½ BDS hormone-free medium under light conditions, the gradual development into polarized, mature embryos was observed (Figure 1o,p). Out of 10 ‘Ornak’ callus clumps subjected to ½ BDS medium, 66 plants were regenerated (Figure 1q,r) after 6–8 subcultures on the same medium. Fifteen out of twenty plants were acclimatized to ex vitro conditions (Figure 1s) as a representative population for flow cytometric analysis. All plants retained the ploidy of donor accession (Figure 1t,u).

### 2.3. Yield and Quality of Embryogenic Callus-Derived Protoplasts

**Figure 2:**
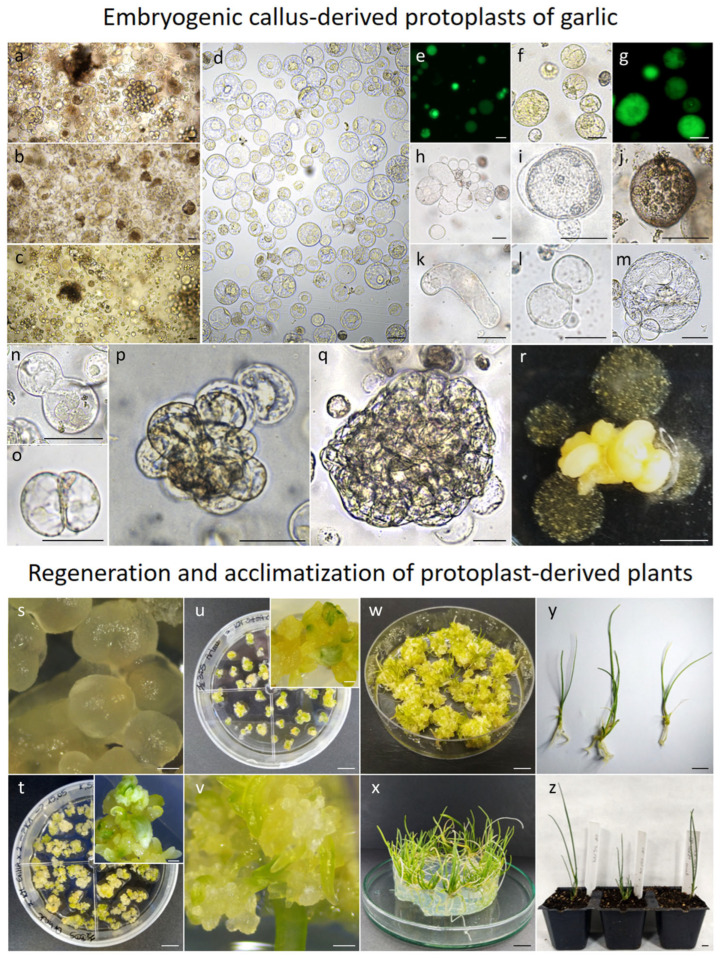
Plant regeneration from embryogenic callus-derived protoplasts in garlic. (**a**–**c**) Freshly released protoplasts of cultivars Arkus, Messidrome and Ornak, respectively, after overnight maceration in HAS+ enzyme mixture and (**d**) after purification steps; green fluorescence of FDA-stained, viable cells (**e**) after protoplast isolation and (**f**,**g**) in 48 h old protoplast culture with noticeable increase in cell size; degeneration and developmental events observed in protoplast cultures: (**h**) cell fragmentation, (**i**) plasmolysis, (**j**) browning or (**k**) elongation, (**l**) stage before reorganization of cytoplasm, (**m**) reorganization of cytoplasm and organelles, (**n**,**o**) re-entering mitotic division; cell colony formation in (**p**) 20- and (**q**) 60-day-old culture; (**r**) somatic embryos and agarose beads overgrown with microcallus; ‘Arkus’ and ‘Ornak’ somatic embryo development (**s**) 1, (**t**,**u**) 2 (insets show a close-up), and (**v**) 3 months after transferring proembryogenic mass to hormone-free ½ BDS medium and light; (**w**) somatic embryos transition into plants after 6 months on ½ BDS medium; (**x**) plantlet growth on U1 medium; (**y**) complete regenerated ‘Ornak’ plants before acclimatization; (**z**) 2-month-old acclimatized ‘Ornak’ plants. Scale bars: 50 µm (**a**–**q**); 1 mm (**r**,**s**,**v**, insets **t**,**u**); 1 cm (**t**,**u**,**w**–**z**).

**Figure 3:**
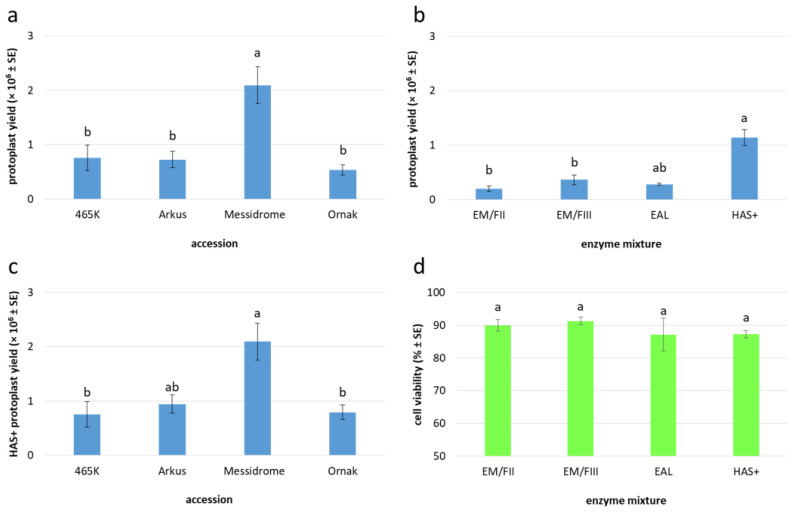
Yield and viability of embryogenic callus-derived protoplasts in garlic. Protoplast yield with respect to (**a**) accession and (**b**) enzyme mixture applied (for details see Table 3); (**c**) effect of HAS+ enzyme mixture on protoplast yield in analyzed accessions; (**d**) effect of applied enzyme mixtures on protoplast viability. Means marked by the same letter within a single plot did not differ significantly at *p* ≤ 0.05 after Tukey’s honestly significant difference test. The Box–Cox transformation was used to normalize the distribution of the dependent variables (protoplast yield or viability).

In general, 10 to 20-day-old garlic embryogenic callus provided a useful source of protoplasts (Figure 2a–c), releasing an average of 0.9 × 10^6^ protoplasts per gram of fresh mass (FM) after purification in sucrose-MES solution (Figure 2d), regardless of the enzyme mixture used for cell wall digestion (Table 3). In total, cultivar Messidrome had the highest yield of protoplasts (approx. 2.1 × 10^6^); for the remaining accessions, the mean yield of protoplasts ranged from 0.5 × 10^6^ to 0.8 × 10^6^ for ‘Ornak’ and 465K, respectively (Table 3, Figure 3a). Of the five tested enzyme mixtures, HAS+ yielded the highest number of protoplasts, averaging 1.1 × 10^6^ cells per gram of callus, regardless of garlic accessions examined, which was more than three times higher compared to the other enzyme mixtures used (Figure 3b). The source accession significantly impacted the yield of protoplasts. In the case of the HAS+ enzyme mixture, the yield varied from 0.8 × 10^6^ to 2.1 × 10^6^ protoplasts per g FM for 465K and ‘Messidrome’, respectively (Figure 3c).

**Figure 4:**
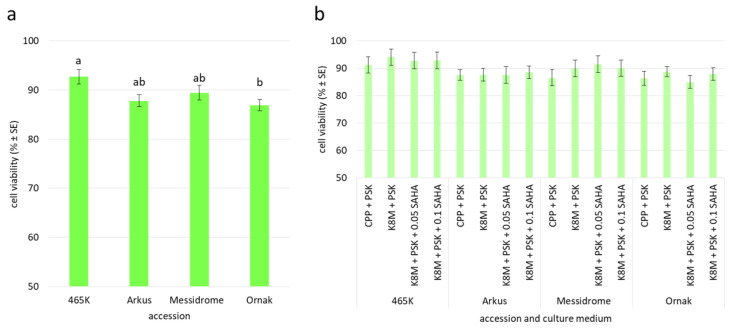
Cell viability determined after 24 h of garlic protoplast cultures with respect to (**a**) accession and (**b**) accession and culture medium variant. Means marked by the same letter within a single plot did not differ significantly at *p* ≤ 0.05 after Tukey’s honestly significant difference test; a plot without statistical annotations (**b**) indicates no statistical differences between the means. CPP—medium acc. Grzebelus et al. [52]; K8M—medium acc. Kao and Michayluk [53]; PSK—phytosulfokine-α (100 nM); 0.05, 0.1 SAHA—0.05 µM or 0.1 µM suberoylanilide hydroxamic acid, respectively.

The quality of the agarose-embedded protoplasts was determined by FDA viability assay approximately one hour after embedding (Figure 2e). The viability of analyzed cells was high regardless of the source accession (ranging from 86% to 89%; Table 3) and the enzyme mixture used for protoplast release (ranging from 87% to 91%; Figure 3d). However, in both cases, the observed differences were not statistically significant. After 24 h of culture, small differences in cell viability with respect to source accession were observed, with values ranging from 87% to 93% for ‘Ornak’ and 465K, respectively (Figure 4a). However, the composition of the culture medium (CPP or K8M) and the application of SAHA (0.05 or 0.1 µM) did not significantly affect cell viability (Figure 4b).

### 2.4. Protoplast Development, Cell Colony and Callus Formation

**Figure 5:**
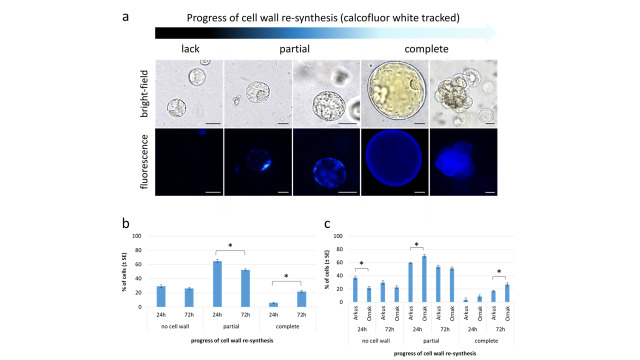
Cell wall re-synthesis in protoplast cultures of garlic. (**a**) Visualization of cell wall re-synthesis from start point of the culture to cell colony formation (light-blue fluorescence indicates cellulose deposition, scale bar: 25 µm); (**b**) progress of cell wall re-synthesis in 24 and 72 h old cultures on K8M + PSK + 0.1 SAHA protoplast medium variant and (**c**) with respect to accession analyzed. Statistically significant differences were calculated using unpaired *T*-test and are marked by asterisks (*p* ≤ 0.05).

One of the first morphological events observed in the protoplast cultures is a change in the perfectly round shape of the cells (Figure 2f–g), primarily due to cell wall re-synthesis, a crucial process in the further development of protoplast cultures. The progress of cell wall reconstruction was monitored for ‘Arkus’ and ‘Ornak’ protoplast cultures in K8M + PSK 0.01 + SAHA medium, at two selected time points, i.e., 24 and 72 h of the culture (Figure 5). At that time, based on calcofluor white cellulose staining, three categories of cells were observed, i.e., (1) those that have not started the cell wall re-synthesis, and (2) have partially or (3) completely restored the cell wall (Figure 5a). After 24 h, over 70% of garlic protoplasts had begun or completely restored their cell wall (65% and 6%, respectively). The re-synthesis process was asynchronous and progressed significantly over time regardless of the cultivar, with 6% of all observed cells with a completely restored cell wall after 24 h, rising to over 21% after 72 h (Figure 5b). A significant change in the number of cells with completely re-synthesized cell walls resulted from a shift from the ‘partial’ to the ‘complete’ category (Figure 5b). No significant change was observed in the number of cells that did not rebuild the cell wall (nearly 30% of all observed cells) between 24 and 72 h of the culture. Some cultivar-specific differences were also observed. Significantly more protoplasts (79%) of ‘Ornak’ began the process of re-synthesis of the cell wall within 24 h of the culture than those of ‘Arkus’ accession (63%). A similar significant relationship was observed in the proportion of cells with completely restored cell wall within 72 h of the culture (Figure 5c).

Further microscopic observations revealed a number of events that occurred during the culture’s duration. The adopted observation model categorized these events as either positive, including pre- and post-mitotic events (e.g., increase in cell size, reorganization of cytoplasm and organelles, re-entry into mitotic division, cell colony formation) or negative (e.g., plasmolysis, cell elongation, fragmentation or browning), indicating development or degeneration of the culture, respectively (Table 4, Figure 2h–q). According to the adopted scale reflecting the frequency of occurring events, much greater accumulation of negative symptoms was observed in the CPP medium compared to the cultures in the K8M medium. These negative events included progressive cell browning during the culture process, as well as the accumulation of cells exhibiting plasmolysis and abnormal elongated morphology. Consequently, mitotic divisions and cell colony formation in CPP + PSK medium variant were strictly limited (Table 4). The K8M as a basal medium partially overcame the abovementioned negative symptoms. Garlic cells increased in size, and on the 20th day of culture, intensive reorganization of cytoplasm and organelles was observed (Figure 2m). Consistently, single cell divisions (Figure 2n,o) and slow cell colony formation (Figure 2p,q) were observed for three out of four garlic accessions (except 465K). At this time point, it was also noticed that the application of SAHA for the first 24 h of the culture had a positive impact on mitotic divisions and cell colony formation for three garlic accessions (i.e., for ‘Arkus’, ‘Messidrome’, and ‘Ornak’). This relationship was even more pronounced in 30-day-old cultures, particularly in the culture variant medium supplemented with 0.1 µM SAHA (Table 4).

**Figure 6:**
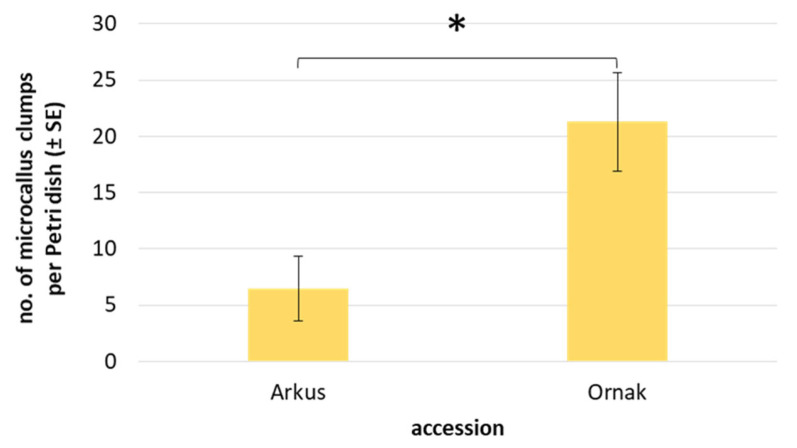
Mean number of microcallus clumps in 90-day-old protoplast cultures of ‘Arkus’ and ‘Ornak’ on K8M + PSK + 0.1 SAHA protoplast medium variant. Statistically significant differences were calculated using unpaired *T*-test and are marked by an asterisk (*p* ≤ 0.05).

The continuous mitotic divisions of protoplast-derived cells led to the formation of cell colonies, which developed into visible microcallus around the 90th day of culture. However, with the exception of a few cases in ‘Messidrome’ and ‘Ornak’ cultures, CPP medium rarely stimulated microcallus formation (Table 5). In contrast, K8M medium stimulated microcallus development in protoplast cultures of three (‘Arkus’, ‘Messidrome’ and ‘Ornak’) out of four tested accessions (Figure 2r). A higher frequency of microcallus formation, particularly evident for ‘Ornak’, was observed in medium variants supplemented with 0.05 µM or 0.1 µM of SAHA for the first 24 h of protoplast cultures. However, such beneficial effect on callus formation was not observed in 465K protoplast cultures (Table 5). Quantitative analysis of microcallus clump development in ‘Arkus’ and ‘Ornak’ protoplast cultures showed that the mean number of callus clumps produced per Petri dish was three times higher for ‘Ornak’ (Figure 6). The transfer of such callus clumps onto a solid K1 medium containing 2,4-D stimulated continuous callus multiplication. After approximately two months of growth, the amount of protoplast-derived callus required for plant regeneration was obtained.

### 2.5. Plant Regeneration and Ploidy Status of Embryogenic Callus Protoplast-Derived Plants

**Figure 7:**
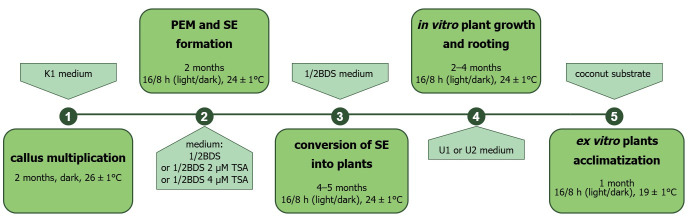
Flow chart illustrating subsequent steps of plant production from protoplast-derived callus of garlic (PEM—proembryogenic mass, SE—somatic embryos, TSA—trichostatin A; for details see Section 4).

Plant regeneration from protoplast-derived callus was carried out for cultivar Ornak. The most effective regeneration-to-rooting approach included ½ BDS medium, which stimulated SE development (from globular to cotyledonary stage) from protoplast-derived callus and U1 medium, which promoted the development of a better root system and faster growth of regenerated plantlets (Figure 2s–y). Medium ½ BDS supplemented with 2 or 4 µM of TSA was tested as a possible factor for accelerating SE formation and consequently reducing the time required for plant development. However, the expected effect was not achieved. The regeneration-to-rooting approach including ½ BDS and U2 media also exhibited a relatively high level of plant regeneration efficiency (approximately 39 plants per dish; Table 6). However, U2 medium did not stimulate root system development and caused vitrification of the plantlets, consequently rendering them completely unsuitable for acclimatization to ex vitro conditions. Regardless of the regeneration-to-rooting approach, the first plants began to develop around 4 months after transferring the callus to the regeneration medium under light conditions. Then the process slowly accelerated, with the peak occurring at around 9 months after starting callus regeneration (Figure 7). In total, 1119 protoplast-derived garlic plants representing cultivar Ornak were obtained and the majority of them (above 75%, with efficiency approx. 54 plants per dish) were produced from protoplast-derived callus induced in K8M + PSK + 0.05 SAHA protoplast medium (Table 6).

Of the 261 plants acclimatized to ex vitro conditions (with more than 64% success rate in total), 76 were subjected to flow cytometry analysis (Table 7). The majority of regenerants obtained via the K8M + PSK + 0.05 SAHA → ½ BDS → U1 regeneration approach were diploid (approx. 97%). However, tetraploids were also identified, accounting for the majority of plants regenerated on ½ BDS medium supplemented with 4 µM trichostatin A (21 from 22 analyzed). Furthermore, three regenerants were characterized by mixed ploidy (2x–4x).

## 3. Discussion

### 3.1. Establishment of an Efficient Embryogenic Callus Induction and Somatic Embryogenesis Protocol for Garlic Regeneration

The present study successfully established an efficient protocol for inducing embryogenic callus from the clove base explants of four garlic accessions, demonstrating regeneration ability through somatic embryogenesis. Koch et al. [57] observed that a high proportion of an inorganic nitrogen source stimulated the formation of friable callus from the base of the garlic clove. Based on these findings, callus induction media proposed in the present study used a B5 micro- and macroelements medium base [58], modified by supplementation with inorganic nitrogen sources in the form of ammonium nitrate and monoammonium phosphate (BDS formula). Based on previous findings by Koch et al. [57] and Luciani et al. [59], induction media were supplemented with either auxin alone or with a combination of auxin and cytokinin to promote callogenesis. For most species, the presence of these plant growth regulators is crucial, as the balance between auxin and cytokinin concentrations significantly stimulates the proliferation and differentiation of embryogenic callus. Higher auxin-to-cytokinin ratios often favor embryogenic over nonembryogenic callus formation [60]. Our findings indicate that the combination of relatively high concentrations of auxins (5 µM of 2,4-D) and cytokinins (9 µM of BAP) is particularly effective in the formation of dry, friable callus but did not lead to a higher frequency of embryogenic callus formation compared to the medium exclusively containing auxins. Although the mean callus formation frequency obtained (56.1%) was lower than that reported by Luciani et al. [59] (88.3%) and Haider et al. [61] (73.5%) for the same type of explant and comparable concentrations of PGRs, it was similar to the frequency reported by Mostafa et al. [62] for shoot tips and leaf explants (45% to 49%). We emphasize that our study, similarly to Mostafa et al. [62], comprised multiple genotypes, and therefore explores genotype specificity often observed in many species [63,64,65]. Moreover, all of the regenerated plants retained the ploidy of donor accession. This demonstrates the potential of the established protocol for widespread application in garlic regeneration studies.

### 3.2. Optimized Protocol for Garlic Protoplast Regeneration: Insights into Embryogenic Callus, Culture Conditions, and SAHA-Mediated Epigenetic Enhancement

The successful regeneration of plants from protoplasts critically depends on the efficient isolation of viable protoplasts, followed by an efficient culture and subsequent differentiation into whole plants. The efficacy of protoplast isolation and subsequent plant regeneration is critically dependent on the characteristics and physiological state of the donor plant tissues, which directly impact both the quantity and viability of the regenerated material [66]. In species recalcitrant to plant regeneration, the use of embryogenic callus may improve regeneration efficiency [21]. On the other hand, callus cultures, particularly those characterized by rapid proliferation, can display somaclonal variation and consequently genetic instability, potentially compromising the fidelity of the obtained plants [67]. Our study shows that the yield (0.8 to 2.1 × 10^6^ cells/g of FM) and quality (86% to 87% of viable cells) of protoplasts obtained from embryogenic callus of garlic are satisfactory for the establishment of protoplast cultures, provided that other crucial factors, such as composition of enzyme mixture, appropriate embedding of protoplasts, and suitable culture medium are carefully adjusted.

Enzymatic digestion of the cell wall requires an optimized enzyme solution tailored to the specific plant material, with most commonly used enzymes including cellulase, macerozyme, pectolyase, and driselase [66]. Our findings demonstrate that a combination of these enzymes, particularly 2% cellulase R-10 and 0.2% pectolyase Y23, is crucial for effective cell wall degradation in garlic callus, leading to a high yield of viable protoplasts. Similarly, the study of Hasegawa et al. [16] demonstrated that a combination of 2% cellulase RS and 0.2% pectolyase Y23 yielded high numbers of protoplasts (up to 9.8 × 10^6^ cells/1 g of FM) from friable callus cultures of garlic. An approximately ten-fold-lower protoplast yield from callus cultures using 1% cellulase RS and 0.1% pectolyase Y23 solution, reported by Ayabe et al. [11], indicates that the concentration of cell wall degrading enzymes was insufficient, or a longer digestion period was required.

The high quality of isolated protoplasts depends on several factors, such as the combination and concentration of enzymes, the method of protoplast embedding, and the composition of culture medium [14]. Although all of the enzyme mixtures used in the present study yielded protoplasts characterized by high viability, we recommend using 2% cellulase R-10 and 0.2% pectolyase Y23 (HAS+ enzyme mixture), as it is the most efficient enzyme combination. Similarly to the work of Ayabe et al. [11] and Hasegawa et al. [16], agarose was used as an embedding agent to avoid cell aggregation and facilitate cell wall re-synthesis and subsequent cell divisions. The formation of multiple small (approx. 30 μL) agarose beads allowed proper aeration and infiltration of culture medium, supplements, and fluorescent dyes.

Protoplast culture medium has a pivotal role in promoting cell divisions and the formation of microcallus. The most effective protoplast culture media vary widely, depending on the species, genotype, and even the source of tissue used. Various protoplast culture media, based on well-established compositions of micro-, macroelements and vitamins (such as MS [57], B5 [52] and KM [54]), and supplemented with various PGRs, have been used for the successful culture of garlic protoplasts [11,16,17,18]. Based on our experience with protoplast cultures of various species, we tested two culture media, both based on KM micro- and macroelements [54], with glucose as a regulator of osmotic pressure and a source of carbon, but differing in their supplementation of PGRs, vitamins, and amino acids. While both media supported initial high protoplast viability, the richer K8M medium consistently promoted positive developmental changes (such as increase in cell size and reorganization of cytoplasm) and consequently more robust cell divisions, leading to microcallus formation.

A key challenge limiting the routine application of somatic embryogenesis in garlic is the low induction rates observed in callus cultures. While certain genes involved in gaining embryogenic competence have been identified, the precise mechanism controlling the entire process remains unknown [68]. Recent research has highlighted the crucial role of reversible changes in histone acetylation in modulating gene expression throughout the process of plant regeneration [69]. Histone deacetylase inhibitors (HDACi) have been demonstrated to boost histone acetylation, thereby modulating a variety of physiological processes [70,71]. Specifically, suberoylanilide hydroxamic acid (SAHA), a well-known histone deacetylase inhibitor, has shown promise in the induction of microspore embryogenesis and enhancement of regeneration efficiency in Pakchoi and ornamental kale by promoting epigenetic modifications conducive to developmental plasticity [41,50]. In this work, we assessed the effect of short exposure of isolated protoplasts to SAHA on culture development, microcallus formation, and subsequently plant regeneration. Short supplementation of culture medium with both 0.05 µM and 0.1 µM SAHA led to increased frequency of positive pre-mitotic events in both 20- and 30-day-old cultures. Exposure to a higher concentration of SAHA promoted cell colony formation in 30-day-old cultures. As a result, more intensive formation of microcallus was observed in 90-day-old cultures of three tested accessions. Interestingly, only callus derived from SAHA-treated cultures displayed regeneration potential, suggesting a pivotal role of this HDACi in the process of protoplast-derived embryo differentiation, development, and regeneration. Our results indicate that a short exposure of protoplasts to HDACi, specifically SAHA, coupled with a continuous presence of PSK, can effectively improve microcallus formation and plant regeneration in garlic. This approach resulted in a 70% success rate of plant acclimatization to ex vitro conditions and retention of the ploidy of the protoplast donor tissue.

## 4. Materials and Methods

### 4.1. Plant Materials

Four accessions of garlic (hereafter referred to as ‘accessions’ representing both gene bank accession and cultivars) with diverse biological and functional properties, reflecting their different genetic backgrounds, were used in the present study (Table 8). The plant materials used in the research came from a gene bank collection (Regional Center for Horticultural Biodiversity, Skierniewice, Poland), resources of Polish breeding company (PlantiCo, Stare Babice, Poland) or were purchased from the market (Benex Company, Hamburg, Germany). All accessions were subjected to callus induction experiments and protoplast isolations and cultures. Histological analyses of induced callus were performed for cultivar Ornak. 

### 4.2. Induction of Callogenesis and Establishment of Stable Callus Cultures

Callus was induced from the base of garlic cloves (Figure 1a,b) as follows: after removing outer skin, cloves were disinfected in 70% ethanol for 1 min and then in a 10% (*w*/*v*) solution of chloramine T (sodium N-chlorotoluene-4-sulphonamide; Chempur, Piekary Śląskie, Poland) with Tween-20 (Duchefa Biochemie, Haarlem, The Netherlands; 125 µL/200 mL of chloramine T) for 20 min. After disinfection, cloves were washed three times (each wash for 5 min) with sterile distilled water. Then the base of the cloves containing the true stem was extracted and cut into four pieces (Figure 1b). Five explants were then placed into a 90 × 25 mm Petri dish (Star™Dish, Phoenix Biomedical, Murcia, Spain) with approximately 25 mL of solid K1 and K2 callus medium (Figure 1c; Table 9). Cultures were maintained at 24 ± 2 °C in the dark. The formed callus was transferred onto fresh medium every four weeks to establish stable callus cultures.

### 4.3. Histological Analysis of Callus and Plant Regeneration from Embryogenic Callus Cultures

Established, eight-month-old callus cultures of cultivar Ornak were used for histological examination. Samples of three to five friable callus clumps (Figure 1g,h) collected from the K1 and K2 solid medium three weeks after subculture, were fixed in freshly prepared 100 mM phosphate buffer (pH 7.2) containing 2% (*v*/*v*) formaldehyde (POCH) and 3% (*v*/*v*) glutaraldehyde (POCH) for at least 48 h at room temperature (with vacuum infiltration within the first 10 min). After fixation, the callus samples were dehydrated in a graded ethanol series (10%, 30%, 50%, 70%, and 90% for 15 min each) and left overnight in absolute ethanol. The dehydrated material was embedded in Technovit^®^ 7100 resin (Kulzer, Hessen, Germany), according to the manufacturer’s protocol. When polymerized, the samples were sectioned into slices of 4 μm thickness using a Leica RM2145 rotary microtome (Leica Microsystems GmbH, Wetzlar, Germany) with a Leica TC-65 carbide blade. The sections were then stained with 1% (*w*/*v*) toluidine blue O (Sigma-Merck, Bavaria, Germany), permanently mounted in Entellan^®^ (Merck), and examined under an Axio Imager.M2 microscope (Carl Zeiss, Göttingen, Germany).

For plant regeneration, clumps of friable callus approximately 1.5 cm in size were transferred into a 90 × 25 mm Petri dish on hormone-free ½ BDS medium (Table 9) and maintained at 26 ± 2 °C with an 18/6 h (light/dark) photoperiod and light intensity of 55 μmol m^−2^ s^−1^. Plant material was subcultured in sterile 500 mL plastic culture vessels (Pakler Lerka, Poland) on the same fresh medium at four-week intervals until fully regenerated plants with a well-developed root system were obtained. The rooted garlic plantlets were then planted in multipots containing moistened coconut substrate (Ceres International Ltd., Pyzdry, Małopolskie, Poland) and placed for ex vitro acclimatization in a SANYO MLR-352H climate chamber (Sanyo Electric Biomedical Co., Ltd., Tokyo, Japan) at 19 ± 2 °C with a 16/8 h (light/dark) photoperiod, a light irradiance of 45 µmol m^−2^ s^−1^ (fluorescent lamps Sylvania Gro-lux T8, Sylvania, Wilmington, MA, USA) and a relative humidity (RH) of 90%. During the four-week acclimatization period, the RH was gradually reduced to 70%, and the plants received moderate watering and were finally moved to a greenhouse at 18–26 °C, a 16/8 h photoperiod, with a light irradiance of 40 µmol m^−2^ s^−1^ (sodium lamps Lucalox LU600W/PSL, Lucalox, Pest, Hungary). The young leaves of acclimatized plants were used for ploidy analysis.

### 4.4. Protoplast Isolation from Embryogenic Callus

Four one-year-old embryogenic callus lines (Table 1) maintained on K1 medium were used as a source of protoplasts. Lines selected for protoplast isolation were subcultured every two to three weeks on a fresh medium to keep callus in good physiological condition. Protoplasts were isolated according to the protocol described by Grzebelus et al. [52] with some modifications. Briefly, 2 g of embryogenic callus (9–20 day-old) was placed in a glass Petri dish (9 cm diameter) containing 8 mL plasmolysis solution (PSII, Table 9), and the callus was cut into small pieces and then incubated for 30 min in the dark at 26 ± 1 °C. Next, the PSII was replaced with one of the tested enzyme mixtures (Table 10) and the samples were incubated overnight (16 h) in the dark at 26 ± 1 °C. For efficient protoplast release from callus, the last 20 min of enzymolysis was carried out with gentle shaking at 50–60 rpm. The released protoplasts were separated from the undigested callus mass by filtering through a 100 µm nylon sieve (Millipore, Burlington, MA, USA), and then centrifuged at 100× *g* for 5 min. The pellet was resuspended in 8 mL of 0.6 M sucrose (POCH, Poland) supplemented with 1 mM MES buffer (2-(N-Morpholino)ethanesulfonic acid; Sigma-Merck), overlaid with 2 mL of W5 solution according to Menczel et al. [72] (Table 9) and centrifuged at 145× *g* for 10 min. The protoplasts localized in the interphase between the sucrose/MES and the W5 solutions were collected using a Pasteur pipette in a new tube and washed twice (first in W5 solution and then in the culture medium) by centrifugation at 100× *g* for 5 min.

### 4.5. Culture of Callus-Derived Protoplasts and Production of Callus Protoplast-Derived Plants

Purified protoplasts were resuspended in 1 mL of culture medium for yield determination using a Fuchs–Rosenthal hemocytometer (Heinz Herenz, Hamburg, Germany) under a light microscope (Leica DM500, Wetzlar, Germany). The working density was adjusted to 8 × 10^5^ cells per ml before embedding the protoplasts in an agarose matrix. For this purpose, an autoclaved solution of 1.2% (*w*/*v*) SeaPlaque agarose (Duchefa) in culture medium was used. Three to four 30 µL aliquots of the protoplast/agarose mixture (at a ratio of 1:1) were dropped into a 6 cm Petri dish. After the agarose beads had solidified (approx. 20 min), 4 mL of the culture medium was added. All protoplast culture media were based on filter-sterilized CPP or K8M medium (Table 9). Four variants of culture media were used: (1) CPP supplemented with 100 nM phytosulfokine-α (PSK; NovoPro, Shanghai, China; CPP + PSK), (2) K8M supplemented with 100 nM PSK (K8M + PSK), (3) K8M supplemented with 100 nM PSK and 0.05 µM suberoylanilide hydroxamic acid (SAHA; Sigma-Merck; K8M + PSK + 0.05 SAHA), and (4) K8M supplemented with 100 nM PSK and 0.1 µM SAHA (K8M + PSK + 0.1 SAHA). After 24 h, the SAHA-containing media were replaced with corresponding SAHA-free media. To prevent bacterial contamination, all protoplast culture media contained 200 mg l^−1^ cefotaxime (Duchefa). Protoplast cultures were incubated at 26 ± 1 °C in the dark. An additional 2 mL of the same culture media was added after 30 days, and if necessary (due to evaporation), after 60 days of the culture.

After approximately three months of protoplast culture, the protoplast-derived microcallus in agarose beads was transferred to K1 medium for callus multiplication. The cultures were maintained at 26 ± 1 °C in the dark and subcultured every three to four weeks for two months. To regenerate plants, the callus clumps with proembryogenic mass (PEM) were transferred to hormone-free ½ BDS medium (Table 9) or supplemented with 2 or 4 µM TSA. Cultures were maintained in a phytotron at 24 ± 1 °C with a 16/8 h (light/dark) photoperiod and a light intensity of 55 µmol m^−2^ s^−1^ (LED FITO PANEL 90 DW + FR, Biogenet, Warszawa, Poland), and subcultured at three-to-four week intervals. After four to five subcultures on regeneration medium, the callus/PEM began to convert into somatic embryos, and subsequently into plants with a weak root system. To develop an extensive root system, the plants were transferred to a rooting medium (U1 or U2, Table 9). Subsequent steps of plant production from protoplast-derived callus of garlic have been summarized in Figure 7. The rooted garlic plantlets were subjected to ex vitro acclimatization as described in the section ‘Histological Analysis Of Callus And Plant Regeneration From Embryogenic Callus Cultures’. The young leaves of acclimatized plants were used for ploidy analysis.

### 4.6. Ploidy Analysis of In Vitro Regenerated Plants

Flow cytometry was applied to determine the ploidy level of garlic plants regenerated from callus cultures derived from both cloves and protoplasts. Samples for analysis were prepared according to the procedure described by Sliwinska et al. [74]. Young and fresh leaves were chopped with a sharp razor blade in a Petri dish containing 1 mL of nuclei isolation buffer (0.1 M Tris; 2.5 mM MgCl_2_·6H_2_O; 85 mM NaCl; 0.1% *v/v* Triton X-100; pH 7.0; all from Merck), supplemented with DAPI (4′,6-diamidino-2-phenylindole; 2 μg/mL; Merck). The nuclei suspension was filtered through a nylon filter with a mesh diameter of 50 μm. For each sample, approximately 2000–2500 nuclei were analyzed, using a CyFlow Ploidy Analyzer flow cytometer (Sysmex Partec GmbH, Münster, Germany) equipped with a linear signal amplification. Histograms were analyzed using a CyView 1.6 software (Sysmex Partec GmbH). Ploidy level was estimated based on the comparison of the position of the G0/G1 peak of the target sample on the histogram with the reference standard which consists of plants of a given cultivar.

### 4.7. Data Collection and Statistical Analysis

In the callus induction experiments, each accession was represented by three biological replications, and each biological replication was represented by 50 explants. Analyses of callus formation and quality were conducted after 18 weeks of continuous culture (three transfers onto fresh medium). Efficiency of callus formation was expressed as the percentage of explants forming callus, whereas callus type and structure were assessed and expressed as the number of explants forming embryogenic vs. nonembryogenic callus (type of callus) and dry and friable vs. watery callus (structure of callus). The mean values and standard errors were calculated based on these assessments.

In experiments involving protoplast cultures, each biological replication was represented by at least three Petri dishes for each treatment. Microscopic observations were carried out on 100–200 cells from at least two dishes per treatment per replication. Protoplast yield was expressed as protoplast number per gram of fresh callus mass (FM) in 1 mL of suspension. Protoplast viability was assessed immediately after embedding the cells in an agarose matrix and after 24 h of culture by staining with fluorescein diacetate (FDA; Sigma-Merk), according to Grzebelus et al. [52]. Viability was expressed as the percentage of cells exhibiting green fluorescence relative to the total number of observed cells. The progress of cell wall re-synthesis was assessed by cellulose staining with calcofluor white M2R (CFW, Merck) after 24 and 72 h of ‘Arkus’ and ‘Ornak’ protoplast culture in K8M + PSK + 0.1 SAHA medium. Briefly, 4 µL of an aqueous CFW solution (0.01% *w*/*v*) was added to the Petri dish with culture of agarose-embedded protoplasts. After 30 min of incubation in the dark, the distribution of cellulose was recognized by blue fluorescence on the cell surface. The progress of cell wall re-synthesis was divided into three classes: (1) no cell wall (no fluorescence signal), (2) partial re-synthesis (blue spots of variable intensity visible on cell surface), (3) complete cell wall re-synthesis (strong blue fluorescence across the entire cell surface). The progress was then expressed as the percentage of cells representing a given class relative to the total number of observed cells. The frequency of developmental and degenerative events was assessed in 20- and 30-day-old protoplast cultures with respect to the culture medium using the following scale: (+++) high, (++) medium, (+) rare, (−) not observed. The assessed parameters included pre-mitotic events (i.e., an increase in size, cytoplasmic and organellar reorganization, unfinished cell divisions), post-mitotic events (formation of cell colonies) and cell degeneration indicators (i.e., plasmolysis, cell elongation, fragmentation and browning).

The frequency of microcallus formation (all callus clumps approx. 1 mm in size) was assessed in 90-day-old protoplast cultures, with respect to the culture medium, using the following scale (shown in Supplementary File S2: Figure S1): (+++) high, (++) medium, (+) rare, (+/−) single events, (−) not observed. Additionally, for the most responsive accessions, i.e., for ‘Arkus’ and ‘Ornak’ cultured in ‘K8M + PSK + 0.1 SAHA’ medium, the number of microcallus clumps was counted and expressed as the mean per Petri dish, with at least three dishes per replication. The efficiency of plant regeneration was assessed by counting the number of fully developed, well-rooted plants and expressed as the mean number of plants per Petri dish. At least three dishes were used for each variant of regeneration-rooting medium. The efficiency of plant acclimation to ex vitro conditions was determined after two months of ex vitro growth and expressed as the percentage of acclimatized plants relative to the total number of planted plants.

All microscopic observations were performed under an inverted DMi8 microscope (Leica Microsystems, Germany) or Axiovert S100 microscope (Carl Zeiss, Germany) with a suitable filter set for fluorescence visualization of fluorescein (λ_Ex_ = 460–500 nm, λ_Em_ = 512–542 nm) and calcofluor white (λ_Ex_ = 320–360 nm, λ_Em_ = 410–450 nm). Statistical analysis was performed for experiments with at least three biological replications. The overall effect of treatments was assessed using analysis of variance (ANOVA) with separation of means performed using Tukey’s post hoc test (HSD) for equal or unequal sample sizes to determine differences between means. Significant differences were expressed at *p* ≤ 0.05. If assumptions of normality (Shapiro–Wilk’s test) and homogeneity of variances (Levene’s test) were not met, the Box–Cox transformation (no. of iterations = 40; −5 < λ < 5; ε = 0.00001) was used to normalize the distribution of the dependent variables (if possible) or the non-parametric Kruskal–Wallis test followed by the post hoc Dunn’s multiple comparison test were used. To determine the statistically significant difference between the means of two groups, an unpaired *T*-test (*p* ≤ 0.05) was used, and statistically significant differences were marked by asterisk. The computations were performed using Statistica ver. 13.0 (StatSoft. Inc., Tulsa, OK, USA).

## 5. Conclusions

This study successfully established and optimized the protocol for embryogenic callus induction and subsequent protoplast-to-plant regeneration in garlic (*Allium sativum* L.), a species historically recalcitrant to such biotechnological advancements. A key finding was the significant role of epigenetic modifiers in enhancing regeneration efficiency. Specifically, short exposure of protoplasts to suberoylanilide hydroxamic acid, a histone deacetylase inhibitor, coupled with long-term exposure to low concentrations of phytosulfokine, proved crucial in improving microcallus formation and plant regeneration. Only callus derived from SAHA-treated cultures demonstrated regeneration potential, highlighting its pivotal function in protoplast-derived embryo differentiation and development in garlic.

The optimized protocol, which also involved fine-tuned enzyme mixtures for protoplast isolation and the use of the enriched K8M culture medium, achieved a 70% success rate for plant acclimatization to ex vitro conditions. Furthermore, 97% of regenerated plants maintained the ploidy of the donor accession, ensuring genetic stability of regenerants. This optimized protocol for garlic protoplast regeneration provides the opportunity for applying emerging genomic tools, such as CRISPR-Cas9, to introduce novel traits into garlic with much needed precision and efficiency.

## Figures and Tables

**Table 1:** Callus formation from base of clove in four garlic accessions and two callus induction media with respect to the structure (friable vs. watery) and type (embryogenic vs. nonembryogenic) of formed callus.

Accession	Callus Induction Medium	Mean No. of Cultured Explants ^1^	Mean No. of Explants Forming Callus ± SE	Structure of Callus (No. of Explants ± SE)		Type of Callus (No. of Explants ± SE)	
				Friable	Watery	Embryogenic	Nonembryogenic
465K	K1	50	40.7 ± 3.5 (81.4%)	0.0	40.7 ± 3.5	31.7 ± 10.7	9.0 ± 7.5
	K2	50	42.7 ± 4.5 (85.4%)	42.7 ± 4.5	0.0	19.0 ± 11.5	23.7 ± 9.9
Arkus	K1	50	24.3 ± 9.4 (48.7%)	22.0 ± 10.5	2.3 ± 1.5	17.3 ± 7.4	7.0 ± 2.1
	K2	50	21.3 ± 3.3 (42.7%)	21.3 ± 3.3	0.0	2.3 ± 2.3	19.0 ± 2.3
Messidrome	K1	50	7.7 ± 0.3 (15.3%)	7.7 ± 0.3	0.0	0.4 ± 0.3	7.3 ± 0.3
	K2	50	13.0 ± 2.1 (26.0%)	13.0 ± 2.1	0.0	4.3 ± 1.7	8.7 ± 0.7
Ornak	K1	50	31.3 ± 6.4 (62.7%)	19.7 ± 6.7	11.7 ± 4.3	20.6 ± 10.8	10.7 ± 5.6
	K2	50	41.3 ± 2.9 (82.7%)	41.3 ± 2.9	0.0	24.0 ± 9.3	17.3 ± 6.5
**Mean**	**–**	**50**	**27.8 ± 3.0 (56.1%)**	**21.0 ± 3.3**	**6.8 ± 2.8**	**15.0 ± 3.2**	**12.8 ± 2.0**

^1^ Each experiment was carried out in three biological replicates, and each biological replicate comprised fifty explants.

**Table 2:** Independent samples Kruskal–Wallis test summary of callus induction for four garlic accessions and two induction media, with respect to the number of explants forming callus, the number of explants forming friable callus, and the number of explants forming embryogenic callus.

Test Summary	Compared Factors					
	Accession and No. of Explants Forming Callus	Induction Medium and No. of Explants Forming Callus	Accession and Friable Callus Formation	Induction Medium and Friable Callus Formation	Accession and Embryogenic Callus Formation	Induction Medium and Embryogenic Callus Formation
Total N	24	24	24	24	24	24
Test statistics (H)	16.06	0.48	4.94	10.90	5.15	1.62
Degree of freedom	3	1	3	1	3	1
Asymptotic significance (*p*)	0.001	0.488	0.176	0.001	0.161	0.203

For significance levels obtained in Dunn’s multiple comparison test, see Supplementary File S1: Tables S1 and S2.

**Table 3:** Isolation efficiency and viability of embryogenic callus-derived protoplasts in garlic (*Allium sativum* L.).

Accession	Enzyme Mixture	Callus Age (Days)	n	Protoplast Yield (×10^6^/g FM) [Mean ± SE]	Protoplast Viability (%) [Mean ± SE]
465K	HAS+	15–20	4	0.75 ± 0.24 (b) ^1^	86.25 ± 3.24 (a)
Arkus	EM/FII	13	1	0.12	94.43
	EAL	11–12	3	0.28 ± 0.02	87.18 ± 5.01
	HAS+	10–16	9	0.94 ± 0.17	86.80 ± 1.92
	**total/mean**	**10–16**	**13**	**0.73 ± 0.15 (b)**	**87.53 ± 1.76 (a)**
Messidrome	HAS+	13–17	6	2.09 ± 0.34 (a)	88.36 ± 2.85 (a)
Ornak	EM/FII	13–16	3	0.23 ± 0.06	88.87 ± 1.74
	EM/FIII	14–17	5	0.36 ± 0.08	91.37 ± 1.20
	HAS+	12–16	7	0.79 ± 0.14	87.61 ± 1.67
	**total/mean**	**12–17**	**15**	**0.54 ± 0.09 (b)**	**89.10 ± 0.89 (a)**
**Total/Mean**		**10–20**	**38**	**0.87 ± 0.12**	**88.69 ± 0.82**

FM—fresh mass; SE—standard error; n—number of independent protoplast isolations (biological replications); ^1^ means within columns with the same letters were not significantly different at *p* ≤ 0.05 after Tukey’s honestly significant difference test was performed for accessions preceded by one-way ANOVA. The Box–Cox transformation was used to normalize the distribution of the dependent variables (protoplast yield and viability).

**Table 4:** Developmental and degeneration events in 20- and 30-day old protoplast cultures of garlic with respect to culture medium.

Types of Developmental/Degeneration Events		Protoplast Culture Medium/Age of the Culture (Days)							
		CPP + PSK		K8M + PSK		K8M + PSK + 0.05 SAHA		K8M + PSK + 0.1 SAHA	
		20	30	20	30	20	30	20	30
pre-mitotic events	increase in size	++	++	++	++	++	++	++	++
	reorganization of cytoplasm and organelles	++	++	+++	+	+++	++	+++	++
	unfinished cell divisions	−	+	+	+	+	+	+	+
post-mitotic events	cell colony formation	−	−	−	++	+	++	+	+++
cell degeneration indicators	plasmolysis	++	++	+	+	+	+	+	+
	cell elongation	++	+++	+	+++	+	+++	+	++
	cell fragmentation	+	++	++	+++	++	++	++	++
	cell browning	++	+++	+	++	+	++	+	++

Table presents summary data for four accessions of garlic, i.e., 465K, ‘Arkus’, ‘Messidrome’ and ‘Ornak’; CPP—medium acc. Grzebelus et al. [52]; K8M—medium acc. Kao and Michayluk [53]; PSK—phytosulfokine-α (100 nM); 0.05, 0.1 SAHA—0.05 µM or 0.1 µM suberoylanilide hydroxamic acid, respectively; frequency of observed events: (+++) high, (++) medium, (+) rare, (−) not observed.

**Table 5:** Microcallus formation in 90-day-old protoplast cultures of garlic with respect to accession and culture medium used.

Protoplast Culture Medium	Accession			
	465K	Arkus	Messidrome	Ornak
CPP + PSK	−	−	−/+	−/+
K8M + PSK	−	+	+	+
K8M + PSK + 0.05 SAHA	−	++	++	+++
K8M + PSK + 0.1 SAHA	−	++	++	+++

CPP—medium acc. to Grzebelus et al. [52]; K8M—medium acc. to Kao and Michalyuk [53]; PSK—phytosulfokine-α (100 nM); 0.05, 0.1 SAHA—0.05 µM or 0.1 µM suberoylanilide hydroxamic acid, respectively; presence of microcallus: (+++) high (more than 8 microcallus clumps/per Petri dish), (++) medium (4–8 microcallus clumps/per Petri dish), (+) rare (1–3 microcallus clumps/per Petri dish), (+/−) single events of microcallus formation, (−) not observed (for details see Supplementary File S2: Figure S1).

**Table 6:** Efficiency of plant regeneration from the protoplast-derived callus of garlic cultivar Ornak.

Protoplast Culture Medium	Regeneration → Rooting Media	n	No. of Obtained Plants	
			Total	Mean/Petri Dish ^1^
K8M + PSK + 0.05 SAHA	½ BDS → U1	15	844	53.6 ± 6.8 (a)
K8M + PSK + 0.05 SAHA	½ BDS + 2 µM TSA → ½ BDS → U1	3	20	6.8 ± 2.9 (b)
K8M + PSK + 0.05 SAHA	½ BDS + 4 µM TSA → ½ BDS → U1	3	45	15.0 ± 1.7 (b)
K8M + PSK + 0.05 SAHA	½ BDS → U2	5	196	39.2 ± 4.2 (ab)
K8M + PSK + 0.1 SAHA	½ BDS → U1	1	14	-

^1^ Efficiency of plant regeneration; n—number of replications (replication = Petri dish), K8M—medium acc. Kao and Michalyuk [53]; ½ BDS—medium acc. to Dunstan and Short [54]; U1—medium acc. to Bohanec and Jakše [55] with modifications (see Section 4, Table 9); TSA—trichostatin A; U2—medium acc. to Murashige and Skoog [56] + 0.5 mg/L NAA; PSK—phytosulfokine-α (100 nM); 0.05, 0.1 SAHA—0.05 µM or 0.1 µM suberoylanilide hydroxamic acid, respectively; means within columns with the same letters were not significantly different at *p* ≤ 0.05 after Tukey’s honestly significant difference test.

**Table 7:** Acclimatization to ex vitro conditions and ploidy analysis of callus protoplast-derived plants of garlic cultivar Ornak.

Protoplast Culture Medium	Regeneration → Rooting Media	Plant Acclimatization (No.)		Ploidy Level (No.)			
		Planted	Acclimatized	Analyzed	2x	4x	2x–4x
K8M + PSK + 0.05 SAHA	½ BDS → U1	183	128 (70.0%)	36	35	0	1
K8M + PSK + 0.05 SAHA	½ BDS + 2 µM TSA → ½ BDS → U1	19	6 (31.6%)	6	5	1	0
K8M + PSK + 0.05 SAHA	½ BDS + 4 µM TSA → ½ BDS → U1	45	22 (49.0%)	22	1	21	0
K8M + PSK + 0.05 SAHA	½ BDS → U2	0.0 ^1^	-	-	-	-	-
K8M + PSK + 0.1 SAHA	½ BDS → U1	14	12 (85.7%)	12	10	0	2

^1^ The plants were not planted because the U2 medium did not stimulate root system development and caused the plantlet vitrification; K8M—medium acc. to Kao and Michalyuk [53]; ½ BDS—medium acc. to Dunstan and Short [54]; U1—medium acc. to Bohanec and Jakše [55] with modifications (see Section 4, Table 9); U2—medium acc.to Murashige and Skoog [56] + 0.5 mg/L NAA; PSK—phytosulfokine-α (100 nM); 0.05, 0.1 SAHA—0.05 µM or 0.1 µM suberoylanilide hydroxamic acid, respectively; TSA—trichostatin A.

**Table 8:** Accessions of garlic used in the present study.

Accession Name	Accession Status/Country of Origin	Bulb Source ^1^	Biological and Functional Characteristics	Experiment Type
465K	gene bank accession/UA	NIHR, PL	early harvest, no other data available	callus induction protoplast cultures
Arkus	cultivar/PL	PlantiCo, PL	medium-early harvest, hardneck winter-hardy cultivar; recommended for direct consumption and processing	callus induction protoplast cultures
Messidrome	cultivar/FRA	Benex, PL	early harvest, softneck winter-hardy cultivar; recommended for direct consumption	callus induction protoplast cultures
Ornak	cultivar/PL	PlantiCo, PL	medium-late harvest, hardneck winter-hardy garlic cultivar; recommended for direct consumption and processing	callus induction histological analysis protoplast cultures

^1^ The National Institute of Horticultural Research, Regional Center for Horticultural Biodiversity, Skierniewice, Poland; PlantiCo—Polish breeding and seed company, Zielonki (Raciborowice), Poland; Benex—Polish flower bulb distribution company, Chrzypsko Wielkie, Poland; FRA—France, PL—Poland, UA—Ukraine.

**Table 9:** Solutions and media used for in vitro garlic cultures including (1) the induction, multiplication and maintenance of callus cultures, (2) callus regeneration, (3) protoplast isolation, culture and plant regeneration.

Solution/Medium Name	Solution/Medium Composition	Application	Storage Conditions
K1 (based on [54] BDS formula)	Gamborg B5 micro- and macroelements with vitamins ([58]; Duchefa Biochemie), 30 g L^−1^ sucrose (POCH, Gliwice, Poland), 320 mg L^−1^ NH_4_NO_3_ (POCH), 230 mg L^−1^ NH_4_H_2_PO_4_ (POCH); 2.0 mg L^−1^ 2,4-dichlorophenoxyacetic acid (2,4-D; Sigma-Merck); 0.6% (*w*/*v*) plant agar (Duchefa); pH 5.8; autoclaved	callus induction and multiplication	room temperature (RT)
K2 (based on [54] BDS formula)	Gamborg B5 micro- and macroelements with vitamins ([58]; Duchefa), 30 g L^−1^ sucrose (POCH), 320 mg L^−1^ NH_4_NO_3_ (POCH), 230 mg L^−1^ NH_4_H_2_PO_4_ (POCH); 1.0 mg L^−1^ 2,4-D (Sigma, St. Louis, MO, USA); 2.0 mg L^−1^ 6-benzylaminopurine (BAP; Sigma); 0.6% (*w*/*v*) plant agar (Duchefa); pH 5.8; autoclaved	callus induction and multiplication	RT
½ BDS ([54])	0.5× Gamborg B5 micro- and macroelements with vitamins ([58]; Duchefa); 30 g L^−1^ sucrose (POCH); 160 mg L^−1^ NH_4_NO_3_ (POCH), 115 mg L^−1^ NH_4_H_2_PO_4_ (POCH); 0.6% (*w*/*v*) plant agar (Duchefa); pH 5.8; autoclaved	plant regeneration	RT
PSII	0.5 M mannitol (Sigma); pH 5.6; autoclaved	plasmolysis	RT
W5 [72]	154 mM sodium chloride (POCH), 125 mM calcium chloride dihydrate (POCH), 5 mM potassium chloride (POCH), 5 mM glucose (POCH); pH 5.8; autoclaved	protoplast purification	RT
K8M medium [53]	KM micro- and macroelements ([53]; Duchefa), 100 mg L^−1^ myo-inositol (Duchefa), 0.01 mg L^−1^ retinyl acetate (Sigma), 1 mg L^−1^ thiamine (Sigma), 0.2 mg L^−1^ riboflavin (Sigma), 1 mg L^−1^ nicotinamide (Sigma), 1 mg L^−1^ D-calcium pantothenate (Duchefa), 1 mg L^−1^ pyridoxine (Sigma), 0.01 mg L^−1^ biotin (Duchefa), 0.4 mg L^−1^ folic acid (Duchefa), 0.02 mg L^−1^ cyanocobalamin (Sigma), 2 mg L^−1^ ascorbic acid (Duchefa), 0.01 mg L^−1^ cholecalciferol, 20 mg L^−1^ sodium pyruvate (Sigma), 40 mg L^−1^ citric acid (Sigma), 40 mg L^−1^ malic acid (Sigma), 40 mg L^−1^ fumaric acid (Sigma), 1 mg L^−1^ choline chloride (Sigma), 0.02 mg L^−1^ p-aminobenzoic acid (Sigma), 68.4 g L^−1^ glucose (POCH), 250 mg L^−1^ sucrose (POCH), 250 mg L^−1^ fructose (Duchefa), 250 mg L^−1^ ribose (Duchefa), 250 mg L^−1^ xylose (Duchefa), 250 mg L^−1^ mannose (Duchefa), 250 mg L^−1^ rhamnose (Duchefa), 250 mg L^−1^ cellobiose (Sigma), 250 mg L^−1^ sorbitol (POCH), 250 mg L^−1^ mannitol (Sigma), 0.6 mg L^−1^ L-alanine, 0.1 mg L^−1^ L-arginine-HCl, 0.1 mg L^−1^ L-asparagine, 0.1 mg L^−1^ aspartic acid, 0.2 mg L^−1^ L-cysteine, 0.1 mg L^−1^ L-cystine, 0.6 mg L^−1^ L-glutamic acid, 5.6 mg L^−1^ L-glutamine, 0.1 mg L^−1^ L-glycine, 0.1 mg L^−1^ L-histidine hydrochloride, 0.1 mg L^−1^ 4-hydroxyproline, 0.1 mg L^−1^ L-isoleucine, 0.1 mg L^−1^ L-leucine, 0.1 mg L^−1^ L-lysine hydrochloride, 0.1 mg L^−1^ L-methionine, 0.1 mg L^−1^ L-phenylalanine, 0.1 mg L^−1^ L-proline, 0.1 mg L^−1^ L-serine, 0.1 mg L^−1^ L-threonine, 0.1 mg L^−1^ L-tryptophan, 0.1 mg L^−1^ L-tyrosine, 0.1 mg L^−1^ L-valine (all amino acids provided by Sigma), 0.1 mg L^−1^ adenine (Sigma), 0.3 mg L^−1^ guanine (Sigma), 0.3 mg L^−1^ thymine (Sigma), 0.3 mg L^−1^ uracil (Sigma), 0.015 mg L^−1^ hypoxanthine (Sigma), 0.03 mg L^−1^ xanthine (Sigma), 0.1 mg L^−1^ 2,4-D (Sigma), 1 mg L^−1^ 1-naphthaleneacetic acid (NAA; Sigma), 0.2 mg L^−1^ zeatin (Sigma), 250 mg L^−1^ N-Z-amine (Sigma), 20 mL coconut water (Sigma); pH 5.6; filtered (0.22 μm membrane; Sterivex-GP, Millipore-Merck, Darmstadt, Germany)	protoplast culture	4 °C, dark
CPP medium [73]	KM micro- and macroelements ([53]; Duchefa), vitamins ([58]; Duchefa), 20 mg L^−1^ sodium pyruvate (Sigma), 40 mg L^−1^ citric acid (Sigma), 40 mg L^−1^ malic acid (Sigma), 40 mg L^−1^ fumaric acid (Sigma), 0.4 M glucose (POCH), 250 mg L^−1^ N-Z-amine (Sigma), 0.1 mg L^−1^ 2,4-D (Sigma), and 0.2 mg L^−1^ zeatin (Sigma); pH 5.6; filtered (0.22 μm membrane; Sterivex-GP, Millipore)	protoplast culture	4 °C, dark
U1 medium (based on [55] formula with modifications)	Gamborg B5 micro- and macroelements with vitamins ([58]; Duchefa), 20 g L^−1^ sucrose (POCH), 15 g L^−1^ maltose (Duchefa), 400 mg L^−1^ myo-inositol (Duchefa), 200 mg L^−1^ proline (Duchefa), 100 mg L^−1^ 2-(N-morpholino)ethanesulfonic acid (MES buffer; Sigma), 320 mg L^−1^ NH_4_NO_3_ (POCH), 230 mg L^−1^ NH_4_H_2_PO_4_ (POCH), 0.5 mg L^−1^ indole-3-butyric acid (IBA; Duchefa), 0.28% (*w*/*v*) gerlite (Duchefa); pH 5.6; autoclaved	plant rooting and shoot growth	RT
U2 medium	MS micro- and macroelements with vitamins ([56]; Duchefa), 20 g L^−1^ sucrose (POCH), 0.5 mg L^−1^ NAA (Duchefa), 0.6% (*w*/*v*) plant agar (Duchefa), pH 5.8; autoclaved	plant rooting and shoot growth	RT

**Table 10:** Composition of enzyme mixtures ^1^ used for protoplast isolation from embryogenic callus of garlic (*Allium sativum* L.).

Component	Enzyme Mixture Name			
	EM/FII	EM/FIII	EAL	HAS+
Cellulase Onozuka R10 (Duchefa)	1%	1%	1%	2%
Pectolyase Y-23 (Duchefa)	-	-	0.04%	0.2%
Macerozyme R-10 (Duchefa)	0.6%	0.6%	0.4%	-
Driselase (Sigma-Merk)	0.15%	0.3%	0.09%	-
Mannitol (Sigma-Merk)	0.6 M	0.6 M	0.6 M	0.55 M
MES buffer (Sigma-Merk)	20 mM	20 mM	20 mM	5 mM
MgCl_2_ × 6H_2_O (POCH)	5 mM	5 mM	5 mM	5 mM
N-Z-amine (Sigma-Merk)	-	-	-	0.1%
pH	5.6	5.6	5.6	5.8

^1^ All enzyme mixtures were sterilized using a 0.22 µm syringe filter and stored in the dark, at 4 ± 2 °C until further use.

## Data Availability

The data underlying this article will be shared on reasonable request to the corresponding author. The plant materials used in the research came from a gene bank collection (Regional Center for Horticultural Biodiversity, Poland), resources of Polish breeding company (PlantiCo) or were purchased from the market (Benex company).

## Supplementary Materials

The following supporting information can be downloaded at https://www.mdpi.com/article/10.3390/ijms27010254/s1.

## Abbreviations

The following abbreviations are used in this manuscript:

2,4-D	2,4-dichlorophenoxyacetic acid
BAP	6-benzylaminopurine
B5	medium base according to Gamborg et al. [57]
FM	fresh mass
HDACi	histone deacetylase inhibitors
IBA	indole-3-butyric acid
KM	medium base according to Kao and Michalyuk [52]
MES	2-(N-morpholino)ethanesulfonic acid
MS	medium base according to Murashige and Skoog [55]
NAA	α-naphthaleneacetic acid
PEM	proembryogenic mass
PGRs	plant growth regulators
PSK	phytosulfokine
SAHA	suberoylanilide hydroxamic acid
SE	somatic embryos
TSA	trichostatin A
