# Supplementary material for: Effect of Suberoylanilide Hydroxamic Acid and Phytosulfokine-Alpha on Successful Plant Regeneration from Embryogenic Callus-Derived Protoplasts of Garlic (Allium sativum L.)
Source: Int J Mol Sci. 2025 Dec 25;27(1):254. doi: 10.3390/ijms27010254 (PMC12785544; doi:10.3390/ijms27010254)
Supplement: Supplementary file 1 [file ijms-27-00254-s001.zip › Supplementary file_S1.pdf]

Supplementary file S1

Table S1. Significance levels obtained in Dunn's multiple comparison test applied to ten tested cultivars with respect to % of explants forming callus.

	Ornak	Arkus	465K	Messidrome
Ornak	—	1	1	0.0173
Arkus	1	—	0.2240	0.6148
465K	1	0.2240	—	0.0012
Messidrome	0.0173	0.6148	0.0012	—

Table S2. Significance levels obtained in Dunn's multiple comparison test applied to two induction media with respect to % of friable callus.

	K1	K2
K1	—	0.0056
K2	0.0056	—
