# Supplementary material for: Effect of Suberoylanilide Hydroxamic Acid and Phytosulfokine-Alpha on Successful Plant Regeneration from Embryogenic Callus-Derived Protoplasts of Garlic (Allium sativum L.)
Source: Int J Mol Sci. 2025 Dec 25;27(1):254. doi: 10.3390/ijms27010254 (PMC12785544; doi:10.3390/ijms27010254)
Supplement: Supplementary file 1 [file ijms-27-00254-s001.zip › Supplementary file_S2.pdf]

Supplementary file S2

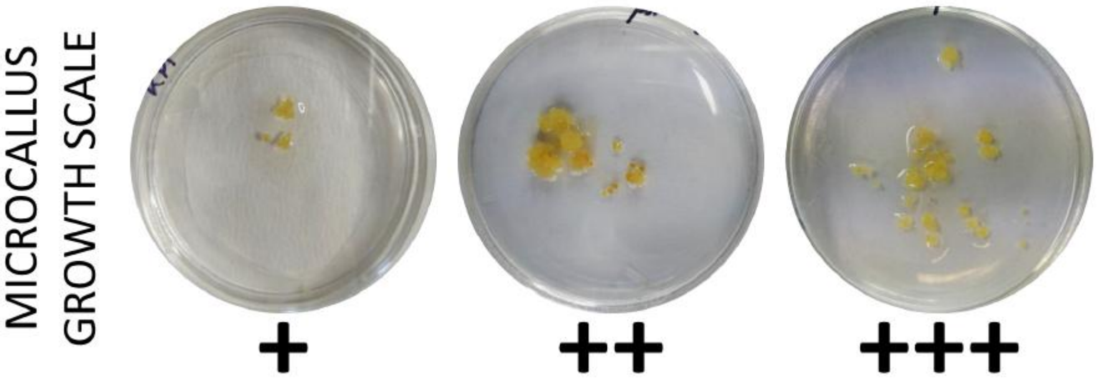

Figure S1. A visual representation of the scale adopted in Table 5 for assessing garlic microcallus formation in 90-day-old protoplast cultures. Presence of microcallus: (+) rare, (++) medium, (+++) high.
